# Neutrophil CD64 index for diagnosis of infectious disease in the pediatric ICU: a single-center prospective study

**DOI:** 10.1186/s12887-022-03738-9

**Published:** 2022-12-15

**Authors:** Lu-Lu Cao, Wei-Wei Wang, Li Zhao, Ji-Ru Li, Xiang-Mei Kong, Yue-Niu Zhu, Xiao-Dong Zhu

**Affiliations:** 1 Department of Pediatric Intensive Care Unit, Xinhua Hospital affiliated to Shanghai Jiaotong University School of Medicine, No. 1665, Kong Jiang Road, Shanghai, 200092 China; 2 Department of Clinical Laboratory, Xinhua Hospital affiliated to Shanghai Jiaotong University School of Medicine, No. 1665, Kong Jiang Road, Shanghai, China; 3 MOE-Shanghai Key Laboratory of Children’s Environmental Health, Xinhua Hospital affiliated to Shanghai Jiaotong University School of Medicine, No. 1665, Kong Jiang Road, Shanghai, China

**Keywords:** Neutrophil CD64 index (nCD64 index), C-reactive protein (CRP), Procalcitonin (PCT), Sepsis, Pediatric intensive care unit (PICU)

## Abstract

**Background:**

Infection is a major cause of death in children, and it is particularly important to identify biological indicators of early infection. Previous studies showed that the neutrophil CD64 (nCD64) index may be a useful biomarker for infection. The purpose of this study was to investigate use of the nCD64 index to identify infection in children from a pediatric ICU (PICU) in China.

**Methods:**

This prospective observational study enrolled 201 children who were admitted to our PICU and were divided into an infection group and a non-infection group. In each patient, C-reactive protein (CRP), nCD64 index, procalcitonin (PCT), and white blood cell count were measured during the first 24 h after admission. Receiver operating characteristic (ROC) analyses were used to determine the sensitivity, specificity, and diagnostic value of the nCD64 index for infection.

**Results:**

Among all 201 children, the infection group had greater levels of CRP, nCD64 index, and PCT (all *p* < 0.05). ROC analysis indicated the nCD64 index had a sensitivity of 68.8%, specificity of 90.7%, accuracy of 80.5%, and an optimal cut-off value of 0.14, which had better diagnostic value than CRP or PCT. For children with postoperative fever, the nCD64 index also distinguished systemic inflammatory response syndrome (SIRS) from infection with accuracy of 79%.

**Conclusions:**

The nCD64 index is a useful biomarker for the diagnosis of early infection in children admitted to the PICU.

**Supplementary Information:**

The online version contains supplementary material available at 10.1186/s12887-022-03738-9.

## Background

Sepsis, severe sepsis, and septic shock are major causes of death in children [1], especially in undeveloped countries. Although the International Consensus Definitions for sepsis and sepsis shock have changed from 1991 (Sepsis-1) to 2016 (Sepsis-3) [2], early diagnosis and antibiotic administration remain the most effective measures to improve prognosis.

For blood stream infections and sepsis, a blood culture is the gold standard for determining the etiology. Enzyme-linked immunosorbent assay, polymerase chain reaction, metagenomic next-generation sequencing, and other methods may also be used for pathogen identification. However, the results from many tests may take 2 to 3 days or more. In addition, blood culture results may be affected by many factors, including previous antibiotic use, sampling time, and contamination [3]. Because of these limitations, blood culture for pathogen detection has only limited clinical usefulness.

To distinguish an infection from systemic inflammatory response syndrome (SIRS) as soon as possible, biomarkers including procalcitonin (PCT), C-reactive protein (CRP), and interleukin-6 (IL-6), may be used. However, the levels of these biomarkers may also be increased in patients with non-infectious conditions, such as surgery, trauma, and other stressors, and in those with non-infection-induced inflammatory responses [4, 5]. Thus, although these biomarkers are widely used in clinical practice, they are not ideal biomarkers.

Neutrophil CD64, a high affinity receptor for the Fc segment of immunoglobulin G, is mainly expressed on the plasma membranes of antigen presenting cells, such as macrophages and monocytes. The expression of CD64 is very low when neutrophils are in a resting state. However, invasion by pathogenic microorganisms can increase CD64 expression by up to 10-fold after 4 to 6 h, following the activation by pro-inflammatory factors [6, 7].

Previous studies suggested that the neutrophil CD64 (nCD64) index may be a sensitive biomarker that can distinguish patients with and without infection [8–11]. Prompt and accurate identification of children with infections can allow appropriate early treatment and improve the prognoses of those with infections, and reduce unnecessary antibiotic use and the social and economic burden of children without infections. The aim of our study was to evaluate the use of the nCD64 index for identification of children in the pediatric ICU (PICU) with and without infection.

## Methods

### Study population

This is a prospective observational study. All children who were 1.3 to 164.2 months-old and admitted to the PICU of our hospital from 1 April to 30 June 2021 were initially examined. When a child was hospitalized two or more times, each hospitalization was recorded separately. The exclusion criteria were: (*i*) neutrophilic deficiency; (*ii*) treatment with a granulocyte stimulating factor in the 2 weeks prior to admission; (*iii*) suspected or confirmed immune deficiency; (*iv*) refusal to participate; and (*v*) no record of the nCD64 index within the first 24 h after admission. A total of 201 children were included in statistical analysis. The clinical treatments of the children were not affected during the study period.

### Collection of clinical data

Demographic and clinical data were collected throughout each patient’s stay in the PICU. White blood cell (WBC) count, CRP, and PCT were measured and different specimen types (sputum, bronchoalveolar irrigation, blood, urine, and wounds) were collected for pathogen detection on the first day of admission.

### Determination of infection

Children in the infection group were classified as having pneumonia, skin and soft-tissue infection, bloodstream infection, digestive system infection, or central nervous system infection. Bloodstream infection was considered to have an unknown origin in children who had no identifiable focus of infection. Digestive system infection includes secondary peritonitis, pancreatitis, and biliary tract infection. Skin and soft-tissue infection includes surgical site infections and necrotizing cellulitis. The diagnostic criteria these different types of infection were from “The International Sepsis Forum Consensus Conference on Definitions of Infection in the Intensive Care Unit” [12].

Central nervous system infection includes bacterial meningitis and viral encephalitis. Briefly, for viral encephalitis, the diagnostic criteria were persistent mental status changes lasting at least 24 h (e.g., mental behavior abnormalities, decreased level of consciousness, personality changes); exclusion of encephalopathy from other causes; and 3 of the following 6 criteria: (*i*) fever (> 38 °C) within 72 h before or after presentation; (*ii*) seizures are not entirely attributable to pre-existing epilepsy; (*iii*) new focal neurological findings; (*iv*) white blood cell count in cerebrospinal fluid of at least 5/μL; (*v*) neurological imaging results suggesting new abnormalities; and (*vi*) EEG abnormalities consistent with encephalitis [13]. Acute viral encephalitis was defined by the presence of a positive virus-specific IgM antibody or a positive polymerase chain reaction test. Bacterial meningitis was defined by: (*i*) fever (> 38.5 °C rectal or > 38.0 °C axillary); (*ii*) headache, meningeal irritation, or altered consciousness; (*iii*) cerebrospinal fluid (CSF) examination showing severe leukocytosis (> 100 × 10^6^ cells/L) or moderate leukocytosis (10–100 × 10^6^ cells/L) with an elevated protein (> 100 mg/dL) or a decreased glucose (< 40 mg/dL); (*iv*) one of three additional criteria (positive CSF culture, positive Gram stain, or positive bacterial antigen in the CSF). A child with the first three criteria was considered to have probable bacterial meningitis; a child with all four criteria was considered to have confirmed bacterial meningitis [14].

### Analysis of nCD64 by flow cytometry

Peripheral venous blood was extracted to determine neutrophil CD64 expression by flow cytometry (BD FACSCanto II, BD, USA). Briefly, 50 μL of peripheral blood was mixed with 20 μL CD45-PerCP and CD64-PE monoclonal fluorescent antibody (BD, USA), followed by gentle vortexing and incubation in the dark for 15 min. Then FACSLysin (1 mL) was added, followed by mixing, and incubation at room temperature away from direct light for 10 min. Then, the cells were washed twice with 2 mL of PBS buffer, suspended in 450 μL of PBS buffer, and examined using flow cytometry. The mean fluorescence intensity of lymphocytes, monocytes, and neutrophils was measured. Based on these fluorescence measurements, the nCD64 index was calculated as: (neutrophils − lymphocytes) / (monocytes − neutrophils).

### Statistical analysis

SPSS version 22.0 (IBM) was used for statistical analysis. Continuous variables were expressed as medians with lower and upper quartiles if the distribution of data was skewed and as means ± standard deviations if the distribution was normal. Categorical variables were presented as numbers and percentages. The *t*-test, *χ*^*2*^ test, or Mann-Whitney U test was used for comparisons, as appropriate.

Receiver operating characteristic (ROC) curves were used to evaluate sensitivity, specificity, and optimal cutoff values of the different biomarkers. Then, a 2 × 2 contingency table was used to evaluate positive and negative predictive value (PPV, NPV), positive and negative likelihood ratios (PLR, NLR), and odds ratios (ORs) with 95% confidence intervals (CIs). Youden’s index was used to identify the optimal cut-off points in the ROC analyses. A two-tailed *p* value below 0.05 was considered statistically significant.

## Results

**Fig. 1:**
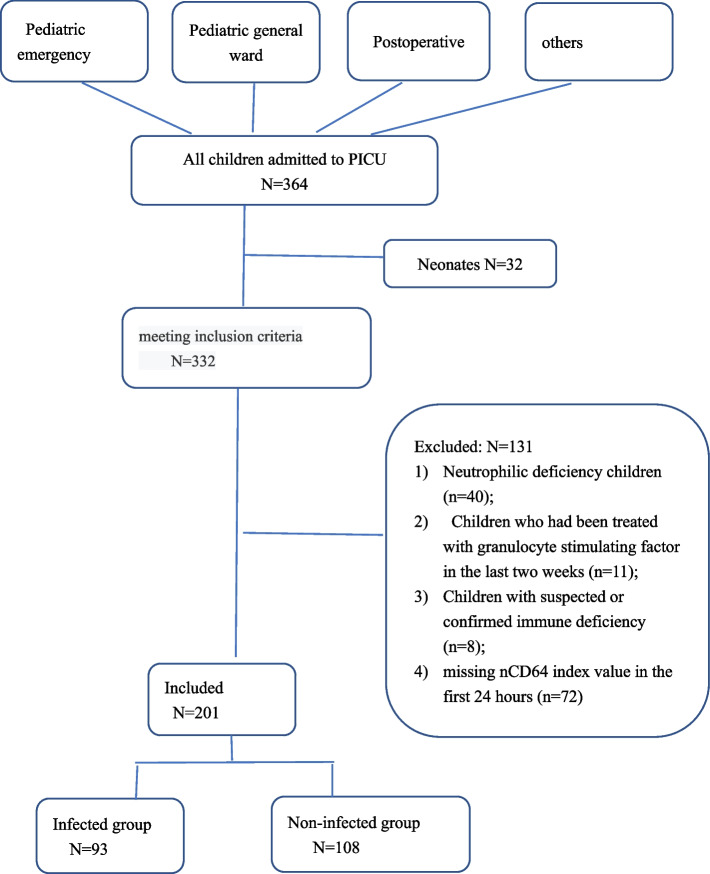
Disposition of patients who were admitted to the PICU (*n* = 364) and were then excluded (*n* = 163), enrolled in the infection group (*n* = 93), or enrolled in the non-infection group (*n* = 108)

A total of 364 children were admitted to the PICU during the 3-month study period, most of whom were from the Pediatric General Ward and Pediatric Emergency Department (Fig. 1). The main reasons for PICU admission were postoperative care and disease progression (deterioration of physical status). Thirty-two of these patients were excluded because they were neonates, and 131 others were excluded based on the predefined exclusion criteria.

We included 201 children in the final statistical analysis, 93 children with infections and 108 without infections (Table 1). Overall, the median age was 49 months, the median length of hospital stay was 15 days, and the median length of PICU stay was 2 days. The infection group was younger, had longer hospital and ICU stays, and was more likely to receive ventilator therapy (all *p* < 0.05), but the two groups had no significant difference in prognosis.

**Table 1:** Baseline characteristics of patients in the two groups at PICU admission

Variable	Infection Group	Non-infection Group	All	***p*** value
	***n*** = 93	***n*** = 108	***N*** = 201	
Age, months	20(6, 58)	67.5(36.25, 96.75)	49(14, 86)	**< 0.001**
Sex				
Male	55 (59.1%)	73 (67.6%)	128 (63.7%)	0.214
Female	38(40.9%)	35 (32.4%)	73 (36.3%)	
Reason for admission				
Disease progression	51 (54.8%)	32 (29.6%)	83 (41.3%)	**< 0.001**
Postoperative care	42 (45.2%)	76 (71.4%)	118 (58.7%)	
Source				
Pediatric ward	16 (17.2%)	1 (0.9%)	17 (8.4%)	**< 0.001**
Pediatric surgical ward	44 (47.3%)	87 (80.6%)	131 (65.2%)	
Pediatric emergency	31 (33.3%)	17 (15.7%)	48 (23.9%)	
Others	2 (2.2%)	3 (2.8%)	5 (2.5%)	
Treatment at PICU				
Mechanical ventilation	31 (33.3%)	8 (7.4%)	39 (19.4%)	**< 0.001**
Hemodialysis/−filtration	6 (6.5%)	5 (4.6%)	11 (5.5%)	0.571
Outcome				
Survival	90 (96.8%)	107 (99.1%)	197 (98.1%)	0.244
Death	3 (3.2%)	1 (0.9%)	4 (1.9%)	
ICU length of stay, days	4(1,9.5)	2(1, 3)	2 (1, 4)	**< 0.001**
Hospital length of stay, days	20 (13, 38)	14 (9, 19)	15(11, 26)	**< 0.001**
nCD64 index	0.18 (0.12, 0.27)	0.09 (0.06, 0.12)	0.11 (0.07, 0.19)	**< 0.001**
PCT, ng/mL	0.19 (0.09,0.83)	0.08 (0.04, 0.22)	0.13 (0.05, 0.31)	**< 0.001**
CRP, mg/L	5 (0.5, 30)	0.5 (0.5, 8)	2 (0.5, 12.5)	**< 0.001**
WBC (× 10^9^/L)	12.16 (8.89, 15.4)	12.29 (8.25, 15.97)	12.16 (8.82, 15.88)	0.0825

Data are indicated as n (%) or median (IQR)

**Fig. 2:**
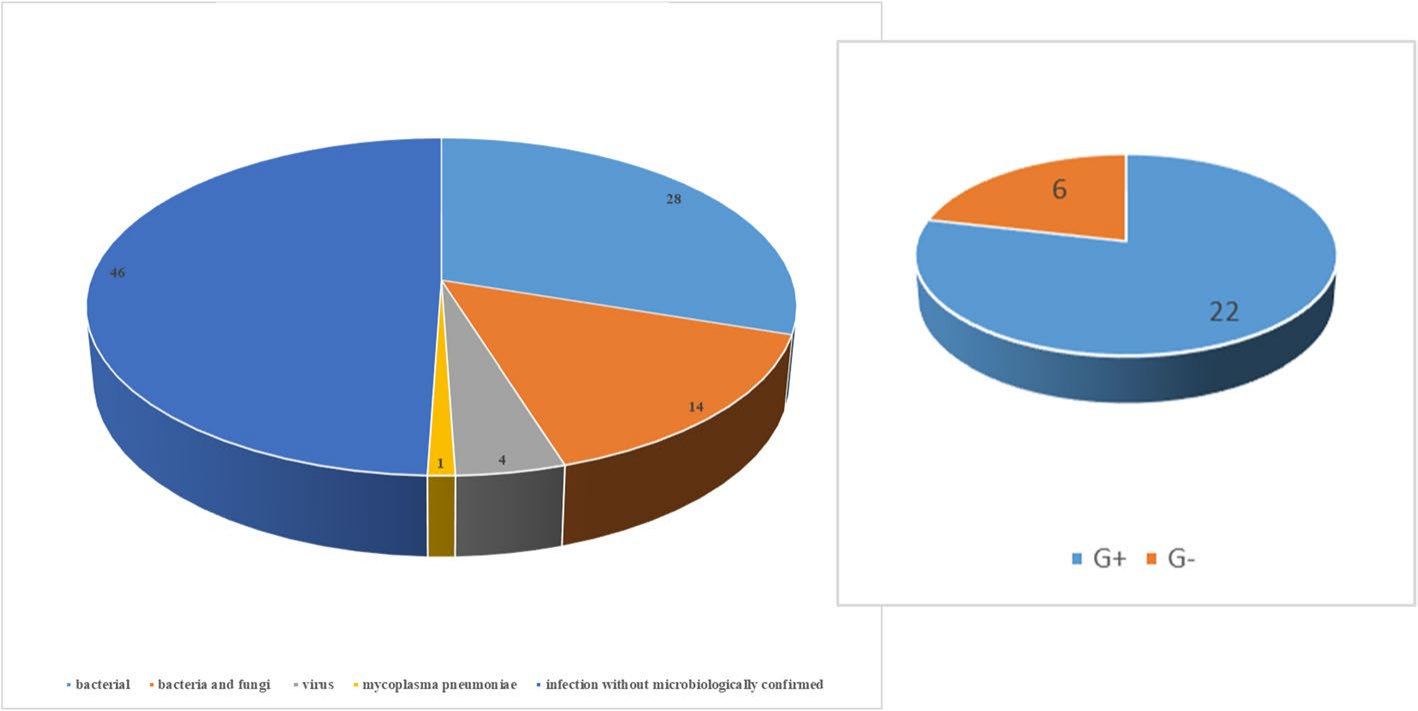
Etiology of infections (left, *n* = 93) and number of Gram-positive and Gram-negative bacterial infections (right, *n* = 28)

**Fig. 3:**
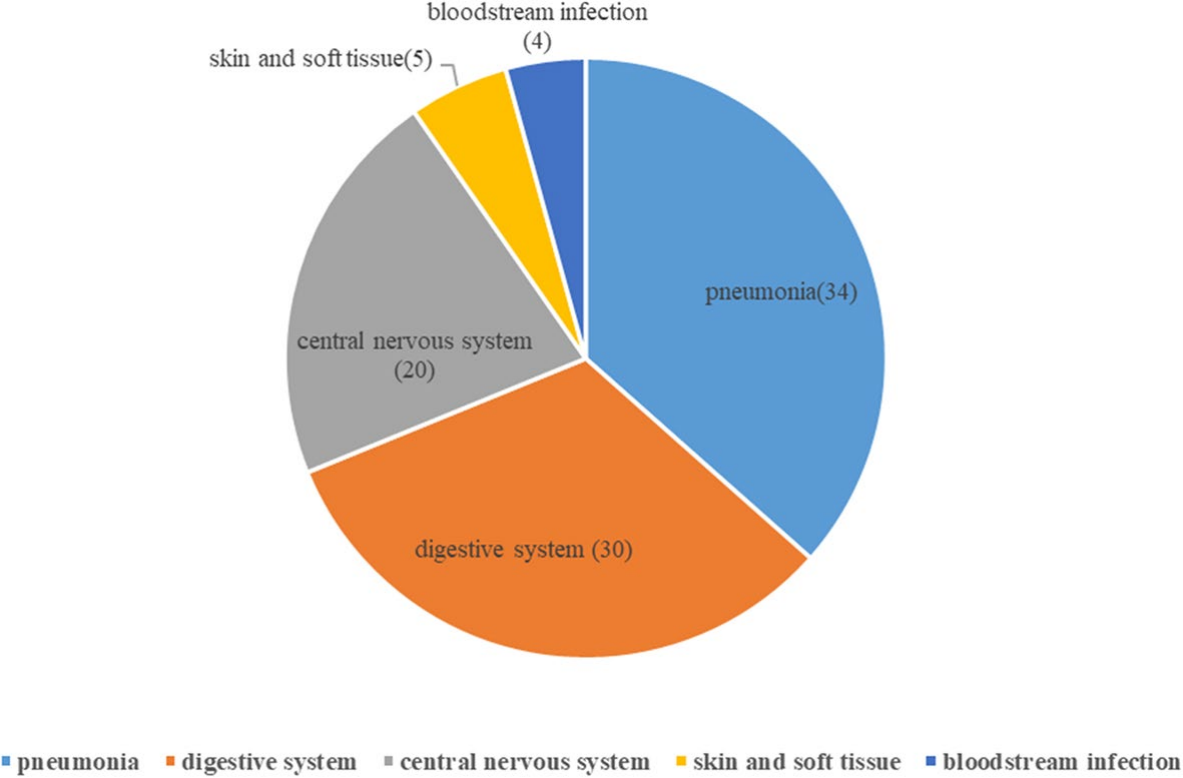
Anatomical sites of infections (*n* = 93)

Among the 93 children in the infection group, pathogenic microorganisms were isolated from the body fluids of the other 47 infected children (Fig. 2). Among patients with microbiological confirmation, 28 were infected by a single bacterial species (22 Gram-positive, 6 Gram-negative), 4 had viral infections, 14 had mixed infections (bacterial and fungal), and 1 had a *Mycoplasma pneumoniae* infection. Among all infected children, 34 had pneumonia, 30 had digestive tract infections (secondary peritonitis: *n* = 26; pancreatitis: *n* = 2; biliary tract infection: *n* = 2), 20 had central nervous system infections (viral encephalitis: *n* = 1; bacterial meningitis: *n* = 19), 4 had bloodstream infections, and 5 had skin soft tissue infections (surgical site infection: *n* = 4; necrotizing cellulitis: *n* = 1) (Fig. 3).

Among children infected with bacteria alone, the isolated pathogens were: *Staphylococcus aureus* (*n* = 15), *Enterococcus faecalis* (*n* = 1), *Enterococcus faecium* (*n* = 1), *Streptococcus pneumoniae* (*n* = 2), *Staphylococcus hominis* (*n* = 1), *Clostridium difficile* (*n* = 1), *Actinomycetes caries* (*n* = 1), *Klebsiella pneumoniae* (*n* = 3), *Klebsiella acidophilus* (*n* = 1), *Escherichia coli* (*n* = 1), and *Haemophilus influenzae* (*n* = 1). The Epstein-Barr virus (*n* = 2), cytomegalovirus (*n* = 1), and adenovirus (*n* = 1) were present in the virus infected group. All fungi in the mixed-infection group were *Candida albicans* (*n* = 14).

**Fig. 4:**
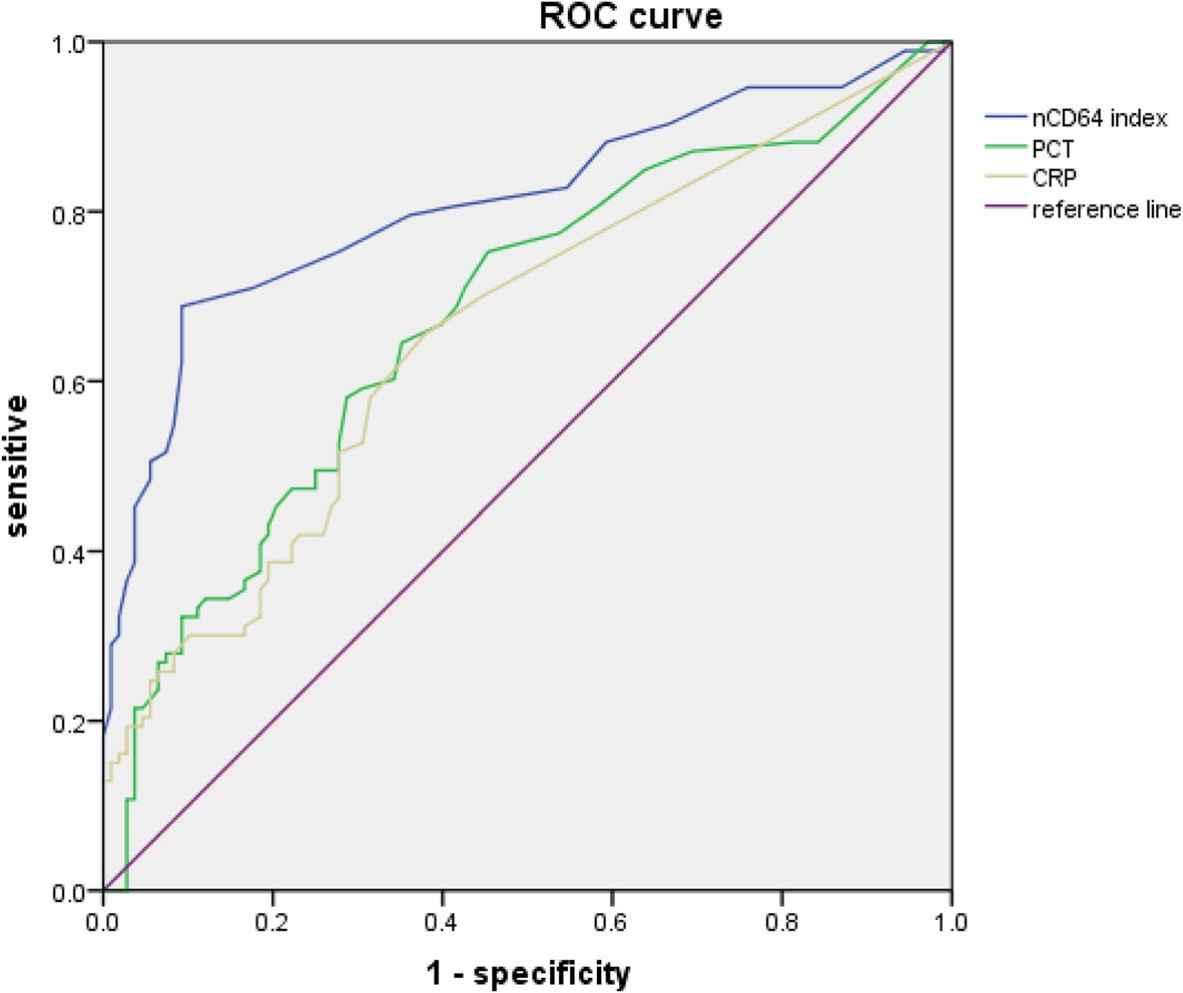
ROC curves for diagnosis of infection based on CRP, PCT, and nCD64 index (*n* = 201)

Comparisons of the different biomarkers in the two groups (Table 1) indicated the infected group had a significantly greater median nCD64 index (0.18 vs. 0.09, *p* < 0.001), median CRP level (5 vs. 0.5 mg/L, *p* < 0.001), and median PCT level (0.19 vs. 0.08 ng/mL, *p* < 0.001). However, the two groups had similar levels of WBCs. We then performed ROC analysis to compare the value of three biomarkers for the diagnosis of infection (Table 2, Fig. 4). For the nCD64 index, the optimal cutoff was 0.14 and the area under the curve (AUC) was 0.811. Pair-wise analysis using Z-test indicated the AUC of CD64 was significantly greater than the AUC values for CRP (0.661, *p* < 0.05) and PCT (0.677, *p* < 0.05). The nCD64 index had a sensitivity of 68.8%, specificity of 90.7%, PPV of 0.86, NPV of 0.77, PLR of 7.4, and NLR of 0.34. Thus, the nCD64 index had greater diagnostic value than CRP and PCT.

**Table 2:** Performance of CRP, PCT, and nCD64 index for diagnosis of infection (*n* = 201)

Biomarker	Cut-off	Sensitivity	Specificity	PPV	NPV	PLR	NLR	Accuracy	AUC (95%CI)
CRP	30 mg/L	76.7%	59.7%	0.77	0.59	1.9	0.39	61.6%	0.661 (0.585–0.736)
PCT	0.5 ng/mL	30%	90.7%	0.72	0.6	3.32	0.7	62.1%	0.677 (0.603–0.752)
nCD64 index	0.14	68.8%	90.7%	0.86	0.77	7.4	0.34	80.5%	0.811 (0.748–0.873)

Postoperative fever is very common in clinical practice, so we analyzed the diagnostic ability of these same biomarkers in the 131 children (87 in the non-infection group, 44 in the infection group) who had postoperative fevers (Table 3). Compared with the non-infection group, the infection group was younger; more likely to receive general surgery, mechanical ventilation, and type II surgical incision; had longer hospital and ICU stays; and had higher levels of the nCD64 index, CRP, and PCT (all *p* < 0.05). All of these children improved and were discharged. Among the children in the infection group, 19 had digestive system infections (secondary peritonitis: *n* = 18; biliary tract infection: *n* = 1), 11 had pneumonia, 9 had central nervous system infections (bacterial meningitis, *n* = 9), 4 had skin and soft tissue infections (surgical site infection, *n* = 4), and 1 case had a blood stream infection. Gram-positive bacteria were the main pathogens (*n* = 16; Supplementary Fig. 1). We also recorded the etiology of the infections in the children with post-operative fevers (Supplementary Fig. 2).

**Table 3:** Baseline characteristics patients who had post-surgical fever

Variable	Infection Group	Non-infection Group	All	***p***
	***n*** = 44	***n*** = 87	***N*** = 131	
Age, months	22.5 (6.5, 63)	66 (34, 97)	16 (13, 24)	**< 0.001**
Sex				
Male	24 (54.5%)	56 (64.4%)	80 (61.1%)	0.343
Female	20 (45.5%)	31 (35.6%)	51 (38.9%)	
Source				
General surgery	28 (63.7%)	31 (35.6%)	59 (45.0%)	**0.001**
Neurosurgery	15 (34.1%)	49 (56.3%)	64 (48.9%)	
Orthopaedic surgery	1 (2.2%)	3 (3.5%)	4 (3.1%)	
Urological surgery	0 (0%)	4 (4.6%)	4 (3.1%)	
Treatment at PICU admission				
Mechanical ventilation				
Yes	11 (25.0%)	3 (3.4%)	14 (10.7%)	**< 0.001**
No	33 (75.0%)	84 (96.6%)	117 (89.3%)	
Hemodialysis/−filtration				
Yes	0 (0%)	1 (1.1%)	1 (0.8%)	0.664
No	44 (100%)	86 (98.9%)	130 (98.2%)	
Type of surgical incision^a^				
I	17 (38.6%)	65 (74.7%)	82 (62.6%)	**< 0.001**
II	23 (52.4%)	13 (15.0%)	36 (27.5%)	
III	0 (0%)	1 (1.1%)	1 (1.1%)	
ICU length of stay, days	3 (1, 5)	1(1,2)	2 (1,3)	**0.001**
Hospital length of stay, days	21 (14.5, 38.5)	14 (11,20)	16 (13, 24)	**< 0.001**
CD64 index	0.15 (0.08, 0.23)	0.09 (0.06, 0.12)	0.11 (0.06, 0.15)	**< 0.001**
PCT, ng/ml	0.17(0.06, 0.55)	0.08 (0.04, 0.2)	0.09 (0.04, 0.23)	**0.005**
CRP, mg/l	2(0.5, 33.25)	0.5 (0.5, 4.0)	0.5 (0.5, 10)	**0.004**
WBC (×10^9^/L)	13.58 (9.59, 17.15)	12.87 (9.7, 17.46)	12.9 (9.7, 17.3)	0.845

Data are indicated as n (%) or median (IQR)

^a^Some children did not receive surgical treatment, so the sum of surgeries is not equal to the total number

We then performed ROC analysis to compare the value of these three biomarkers for the diagnosis of infection in children who had post-operative fevers (Table 4). The results indicated the nCD64 index had a sensitivity of 56%, a specificity of 90%, PPV of 0.73, NPV of 0.81, PLR of 5.6, and NLR of 0.49. Pair-wise analysis using the Z test indicated the AUC for the nCD64 index (0.722) was significantly greater than the AUC values for CRP (0.641, *p* < 0.05) and PCT (0.649, *p* < 0.05).

**Table 4:** Performance of CRP, PCT, and nCD64 index for diagnosis of infection in patients who had post-surgical fever (*n* = 134)

Biomarker	Cut-off	Sensitivity	Specificity	PPV	NPV	PLR	NLR	Accuracy	AUC (95%CI)
CRP	30 mg/L	27%	93%	0.67	0.72	3.8	0.78	71%	0.641 (0.537–0.744)
PCT	0.5 ng/mL	25%	98%	0.85	0.72	12.5	0.77	73%	0.649 (0.546–0.752)
nCD64 index	0.14	56%	90%	0.73	0.81	5.6	0.49	79%	0.722 (0.621–0.823)

## Discussion

The purpose of this prospective study was to investigate use of the nCD64 index as a biomarker for infection in children admitted to the PICU. Our results confirmed that the nCD64 index effectively distinguished children with and without infections, and also had better diagnostic performance than PCT and CRP. To reduce the influence of stress, surgery, and other factors on these biomarkers, we included children in postoperative care, children admitted to the PICU because of emergency, and children transferred to the PICU from the general ward. Thus, our results provide real-world evidence that the nCD64 index can distinguish infected and non-infected children who were admitted to the PICU.

We found that the nCD64 index had a sensitivity of 68.8%, specificity of 90.7%, and accuracy of 80.5% for the diagnosis of infection at PICU admission. This suggests that the nCD64 index is a useful biomarker of infection in these pediatric patients. Our results were similar to a previous 2007 study [15]. Dal Ponte et al. studied 12 patients with SIRS, 45 with sepsis, and 52 with suspected sepsis, and measured the nCD64 index and other sepsis biomarkers within 6 h of hospital admission and after 48 h of hospitalization. They found that the nCD64 index differentiated sepsis from SIRS with an accuracy of 82.1% [16]. A meta-analysis concluded that the nCD64 index had better diagnostic value for sepsis than PCT and IL-6 [8]. A 2021 prospective cohort study of a PICU in China that examined 335 children suggested that nCD64 index was valuable for the early diagnosis of sepsis and reliably predicted the prognosis of children with sepsis [9].

We also performed an analysis of postoperative children who had fevers prior to PICU admission. Postoperative fever is common in children, and it is important to determine whether this fever is caused by surgical stress or infection. Our subgroup analysis suggested that the nCD64 index reliably distinguished infected and non-infected children in this subgroup of postoperative children with fevers. A clinical trial by Vicente López et al. reported similar results [17]. They showed that the nCD64 index was a reliable biomarker for infection in patients with postoperative fever, with a sensitivity of 56% and a specificity of 90%. Other research that compared different infection indexes, such as PCT and CRP, also found that the nCD64 index was a reliable marker of postoperative infection [18].

A previous study suggested that the nCD64 level reliably distinguished bacterial infection from viral infection. In particular, for children admitted to an emergency department with fever, the nCD64 level was higher in an infection group than in a non-infection group; within the infection group, nCD64 expression was higher in the group with bacterial infection than viral infection [19]. The children in our infection group included those who were infected with bacteria, viruses, mycoplasma, and fungi. Due to the small number of our children infected with viruses alone (*n* = 4), we could not meaningfully analyze use of the nCD64 index to distinguish bacterial and viral infections.

Many recent studies examined use of CD64 as a marker of infection. The results suggest that CD64 has advantages in the diagnosis of infection, but these many studies have used different specific indicators, such as nCD64, nCD64 index, and CD64 mean fluorescence intensity (MFI) [20]. Similar to the nCD64 and CD64 MFI, the CD64 considers the expression of CD64. However, the nCD64 index is less affected by exogenous factors (such as instrumental fluctuations) and subjective factors. In addition, although nCD64 index and the nCD64 MFI can be used as biomarkers to distinguish SIRS from sepsis in critically ill children, the nCD64 index appears to be superior to these other biomarkers in clinical diagnosis [21]. We therefore focused on the nCD64 index in this study.

Our results are consistent with the results of a study of PICU patients by García-Salido et al. [22]. who measured nCD64 MFI as a diagnostic indicator of infection. Similarly, an investigation conducted in a NICU showed that the nCD64 MFI provided a reliable diagnosis of neonatal sepsis, with a sensitivity of 85.6%, a specificity of 93% and a cutoff of 43% [23].

Although previous studies have established that nCD64 is a rapid and simple biomarker of infection, they have used different evaluation methods, and the cutoff values were therefore also different. In particular, Thiriet C et al. used the nCD64 index to diagnose sepsis and their cut-off value was 0.48 [20], Dal Ponte et al. used the nCD64 index to diagnose sepsis and their cut-off value was 1.45 [16], and we used the nCD64 index to identify infection in the PICU and our cut-off value was 0.14. Notably, our cut-off value of 0.14 is very similar to that reported in two previous studies [24, 25].

Our ROC analysis confirmed the nCD64 index provided reliable prediction of infection and also distinguished SIRS from infection in children with postoperative fever. However, our study was limited in that it was an observational study conducted at a single center in China. Thus, the value of the nCD64 index as a biomarker for the diagnosis of early infection in children admitted to the PICU needs to be confirmed in different populations and in multi-center studies. There are several additional topics that should also be addressed in further studies, such as the methods for measurement of the nCD64 index, use of the nCD64 index rather than nCD64 MFI, and the optimal cut-off value for the nCD64 index.

## Conclusion

The major findings of this prospective observational study of 201 children who were admitted to our PICU were that the nCD64 index at admission can be used to identify early childhood infection, and it provided greater diagnostic value than CRP or PCT.

## Supplementary Information

**Additional file 1: Supplementary Figure 1.** Sites of infections in patients who had post-surgical fever (*n* = 44). **Supplementary Figure 2.** Etiology of infections in patients who had post-surgical fever (*n* = 44). For the 17 children with bacterial infection alone, there were 16 Gram-positive species (*Staphylococcus aureus* [*n* = 12], *Enterococcus faecium* [*n* = 1], *Enterococcus faecalis* [*n* = 1], *Streptococcus pneumoniae* [*n* = 1], *Staphylococcus hominis* [*n* = 1]) and 1 Gram-negative species (*Haemophilus influenzae*).

## Data Availability

The datasets generated during and analyzed during the current study are not publicly available now because the follow-up observation test is still in progress; but are available from the corresponding author or first author on reasonable request.

## Abbreviations

nCD64: Neutrophil CD64
CRP: C-reactive protein
PCT: Procalcitonin
ROC: Receiver operating characteristic
SIRS: Systemic inflammatory response syndrome
IL-6: Interleukin-6
PPV: Positive predictive value
NPV: Negative predictive value
PLR: Positive likelihood ratios
NLR: Negative likelihood ratios
ORs: Odds ratios
CIs: Confidence intervals
