# Supplementary material for: Neutrophil CD64 index for diagnosis of infectious disease in the pediatric ICU: a single-center prospective study
Source: BMC Pediatr. 2022 Dec 15;22:718. doi: 10.1186/s12887-022-03738-9 (PMC9753391; doi:10.1186/s12887-022-03738-9)

Supplementary Figure 1. Sites of infections in patients who had post-surgical fever before PICU admission (n = 44).

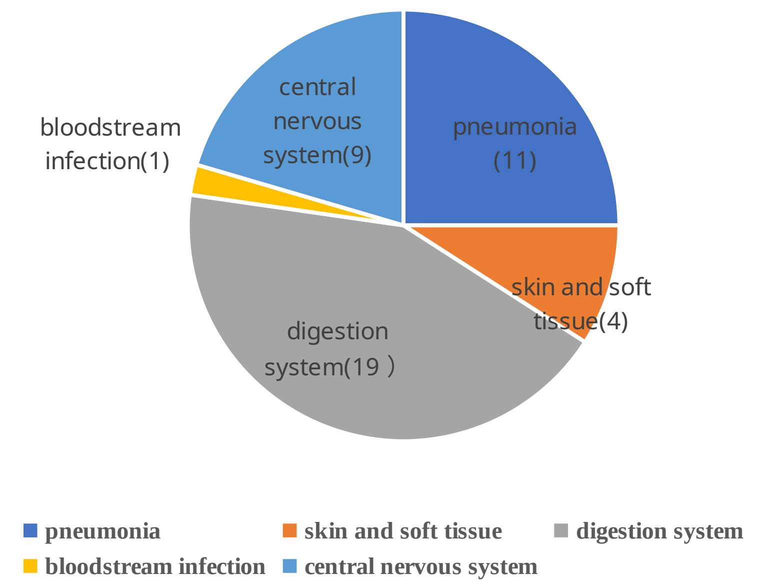

Supplementary Figure 2. Etiology of infections in patients who had post-surgical fever (n = 44).

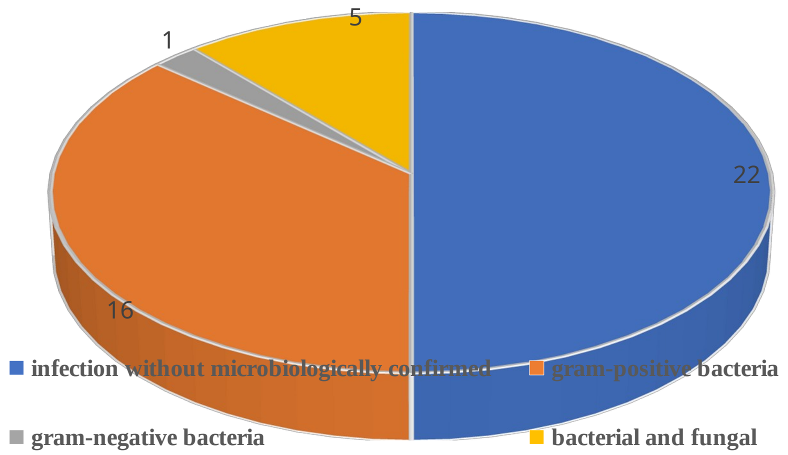

Supplement: Supplementary file 1 [file 12887_2022_3738_MOESM1_ESM.docx]
